# Household air pollution related to biomass cook stove emissions and its interaction with improved cookstoves

**DOI:** 10.3934/publichealth.2021024

**Published:** 2021-03-25

**Authors:** Rebecca Pratiti

**Affiliations:** Department of Internal Medicine, McLaren Health Care, USA

**Keywords:** indoor air pollution, cookstove, particulate matter, polycyclic aromatic hydrocarbons, carbon monoxide

## Abstract

**Introduction:**

Household air pollution (HAP) is associated with significant global morbidity and mortality. Newer initiatives including improved cookstove (IC) and cleaner fuels are being implemented to improve HAP effects.

**Methods:**

A literature review was conducted for household air pollution related to biomass cookstoves in resource limited countries. In January 2018, we electronically searched the PubMed database for the term cookstoves with no date restrictions. We included cohort, case-control, cross-sectional studies, conference abstracts, editorials, and reviews; studies that assessed the emissions related to cookstove and factors affecting HAP emissions.

**Results:**

Twenty-three articles met the objectives of the review. Fine particulate matter with aerodynamic diameter <2.5 µm (PM_2.5_), carbon monoxide (CO) and polycyclic aromatic hydrocarbons (PAH) are the major HAP emissions. Emission factors are based on the stove and fuel used while the activity is based on cooking practices. Changes in composition and sources of PM_2.5_ causes modification to its resulting toxicity. Many PAHs and their metabolites released by HAP have carcinogenic, teratogenic and mutagenic potential. Improving ventilation decreases concentrations of PM_2.5_ and CO in the household air. Few standard tools are available to measure ventilation and continued IC efficacy in long term.

**Conclusion:**

Unavailability of tools to measure ventilation and continued IC efficacy in long term affect uniformity and comparability of IC study results. Community education about the health effects of HAP and importance of ventilation in decreasing HAP is an important aspect of public health policy to prevent HAP effects.

## 1. Introduction

Almost three billion people are exposed to household air pollution (HAP) through cooking with biomass based cookstoves [1]. Two thirds of the HAP exposure risk population reside in South East Asia and Africa [2]. Some of the major areas with high HAP exposure occurs near Smoky coal mines in China and Indo-Gangetic plains near Himalayas in India [3],[4]. Further, a study on global atmospheric impacts of residential fuel burning found emissions like PM_2.5_, organic compounds and black carbon are dominated by transport in North America, by open burning in Africa and by residential solid fuels in Asia [5]. An estimated 2.6 million premature deaths are attributed to HAP [2],[6]. Additionally, HAP caused 5 million deaths annually and 110 million disability-adjusted life years (DALYs) in 2010 [7]. As per Global Burden Disease (GBD) Assessment, 3–5% GBD in terms of DALY is caused by HAP with almost two thirds occurring in children less than 5 years and the rest in the adult population. Health effects associated with HAP mostly include increased blood pressure (BP), dyspnea, childhood pneumonia, lung cancer, low birthweight and cardiovascular diseases [6],[8]. This exposure and health effects is highest for the vulnerable population of women and children resulting in significant cumulative health effects. More recently, emission including particulate matter exceeding safe levels has been associated with higher number of coronavirus disease 2019 (COVID-19) infection and mortality. This pollution could also be conducive to the transmission of severe acute respiratory syndrome coronavirus 2 (SARS-CoV-2), which is the strain of novel coronavirus that causes COVID-19 [9].

Most HAP health effects are caused by emissions of fine particulate matter with aerodynamic diameter PM_2.5_, CO and PAH. Research had shown interventions decreasing emissions without necessarily attaining WHO target results in 20% to 50% decrease in risk of several health-related outcomes [10]. HAP is a global problem with divergent environmental factors including wide variety of fuel used, housing condition, foods prepared, climatic condition and social factors; most solutions for HAP though efficient seems inadequate. This heterogeneity in cooking practices affects the outcome assessment of improved cookstove health assessment.

The data is more heterogenous for childhood pneumonia. Studies like Randomized exposure study of pollution indoors and respiratory effects (RESPIRE) and Arlington et al. showed benefit for preventing childhood pneumonia, on the other hand studies by Mortimer et al and Clark et al. did not show benefit [11]–[14]. Similar diverse results are seen in IC studies evaluating birthweight and pregnancy outcomes. Thus, the health outcome mitigation by IC in various studies is divergent [6]. The review assessed the factors for these diverse results.

## 2. Study design

### 2.1. Research setting

With the significant health burden caused by HAP and the economic limitations of switching completely to cleaner fuels; interventions are being focused on improved cookstove (IC). One of the initiatives is the Global Alliance for Clean Cookstoves. Improved cookstove (IC) mitigates emissions and improves short term health, though few randomized studies have studied the effect of HAP emission factors on IC. IC were developed to improve fuel efficiency and ventilation during cooking. Improved cookstoves have decreased personal exposure to PM_2.5_, CO and PAH [15]. Challenges to adoption of cleaner fuels and IC include cost feasibility, stove sustainability, fuel access and cultural acceptance [16]. The objectives of the review were to assess the HAP related emissions, factors affecting these emissions and how the interaction of these factors affect IC related study design and outcomes.

### 2.2. Data sources

For the literature review, In January 2018, we electronically searched the PubMed database for the term cookstove, with no date restrictions. Studies without English translation were excluded. Cookstove was not available as a MeSH term.

### 2.3. Variables

Study inclusion and exclusion criteria: We included (1) cohort, case-control, cross-sectional studies, conference abstracts, editorials and reviews; (2) studies that assessed the emissions related to cookstove (3) studies that assessed factors affecting HAP emissions (4) studies that assessed health effects of HAP. We excluded case reports and studies related to ambient air pollution. All data cited confirmed to Helsinki Declaration on human experiments.

### 2.4. Logic of investigation

Article selection, data extraction and analysis: Articles were reviewed, and eligible articles were selected based on the selection criteria outlined above. Articles were included after initial scanning of the titles and abstracts. If the criteria were not easily identifiable from the titles and abstract, a full article review was then conducted to confirm eligibility.

Study titles and abstracts were screened, and 23 articles met the objectives of the review. Full texts of these articles were downloaded and reviewed further. This project was done as part of project to assess emissions related to HAP caused by biomass cookstoves and their effects on health. The HAP emission health effects have been reported elsewhere [6]. This review is to focus on the HAP related emissions and factors affecting HAP emissions.

No statistical data analysis was performed as a part of this study.

## 3. Results and discussion

We found solid fuel combustion for cooking has significant health and environmental impacts. Household air pollution related to cooking is henceforth termed HAP. The major HAP emissions are PM_2.5_, carbon monoxide and polycyclic aromatic hydrocarbons.

### 3.1. Particulate matter

PM_2.5_ levels showed substantial variability with sparse measurement. Factors contributing to these variabilities included seasonal variation, with higher levels in winter and changes in day-to-day cooking patterns. Changes in the composition and sources of PM_2.5_ caused modification to its resulting toxicity, mutagenicity and oxidative potential [17]. Because of the low combustion efficiencies, with high consumption amounts of traditional fuels like wood, coal and agricultural wastes, nearly half of primary PM_2.5_ were from residuals of solid fuel combustion [18],[19].

PM_2.5_ is the best measure of HAP exposure and the most predictive in relation to health [17]. Open ﬁre cookstoves using biofuels emit approximately 73% more PM_2.5_ than improved stoves with a ventilation system [14]. WHO recommendation for mean PM _2.5_ for a 24-h period is no more than 25 µg/m^3^. Biofuel use in multiple homes within a community could increase the ambient PM_2.5_ levels above this limit in some regions [20].

It would be more beneficial to have PM_2.5_ analyses including data on composition and source apportionment [17]. PM_2.5_ concentration measurement should be done in the cooking area, in the primary cook and other people of the household. Studies on HAP quantifying PM_2.5_ concentrations during cooking should adjust for baseline ambient concentration [21]. For this, studies could include baseline outdoor PM_2.5_ and personal and indoor PM_2.5_ measurement during non-cooking hours that may possibly affect the outcome measures of IC [22]. Less intra-person and inter-person variability for PM_2.5_ is noticed when more frequent PM_2.5_ monitoring is done [23]. In summary, PM_2.5_ is an important objective marker of HAP related health and its mitigation by IC.

### 3.2. Carbon monoxide (CO)

CO measuring methods for evaluating indoor air pollution were cheaper and easier to use than PM-monitoring devices. Hence, CO measurements were used in HAP studies as a proxy for PM_2.5_ [24]. Though CO and PM_2.5_ had good correlation, their reported correlations varied across settings. The largest correlations were noted where both pollutants were largely derived from biomass burning, and lower correlations were observed in rural settings and in settings with probable multiple sources of pollution [25]. Similarly, the indoor and personal CO measurements corelated highly with each other making it less cumbersome for study participants to wear the monitor [22]. A study comparing cookstoves, showed when 1 kilogram of dung is burnt containing 334 g carbon, 26.35 g of carbon in the form of CO is released. On the other hand, if a cleaner fuel like 1 kg of biogas containing 396 g of carbon is used, it releases 0.836 g of carbon as CO. Consequently, CO release was significantly decreased with cleaner fuels [26].

WHO indoor air quality standards recommend CO concentration below 6 parts per million (ppm) over 24 hour period [27]. In a study from Ethiopia, the CO levels were higher than WHO guidelines [22]. Traditional cookstoves are considered the largest sources of black carbon which is responsible for global warming [26]. Hence replacing traditional cookstoves would also lead to increased protection for climate and health. Though IC have shown decrease in CO release from 30% to 70% depending on the type and mechanism of the cookstove, the CO emissions remains higher than the WHO recommended limit. This signifies the importance of fuel efficiency [28]. Short term exposure to CO is associated with respiratory and cardio-vascular morbidity and mortality. Long-term exposure to CO is associated with negative birth outcomes, developmental effects and central nervous system effects [6].

Recent systematic reviews and meta-analysis have evaluated personal and kitchen environment CO levels in households with traditional cookstoves as compared to IC. The data showed that both the personal CO levels and kitchen CO levels are reduced in the presence of newer cookstoves except studies scoring low in the Effective Public Health Practice Project Quality Assessment Tool (EPHPP). The metanalysis showed substantial heterogeneity but there was no evidence of the small study effect which was conﬁrmed further by the trim and ﬁll method [15]. HAP related CO levels are a function of activity and emission factors. Emission factors are based on the stove and fuel type used while the activity is based on cooking practices, such as the timing, duration of cooking and the types of dishes prepared [29]. These factors are more difficult to control for in a randomized cookstove study and hence significant heterogeneity is noticed in systematic review or meta-analysis of cookstove studies. Consequently, most cookstove studies are designed as pre-post study design [6]. Only few studies have reported kitchen exposures not corelating with personal CO exposures by 30–40% that may be related to signiﬁcant environmental air pollution [4]. Nonetheless, CO is an easy to measure objective biomarker of HAP that is independent of outdoor ambient environmental pollution and non-cumbersome to study participants.

### 3.3. Polycyclic aromatic hydrocarbons (PAH)

PAH were a subset of organic compounds formed as a result of pyrolysis or inefficient combustion of organic material. These were stable compounds containing carbon and hydrogen with two or more fused aromatic rings [30]. These compounds existed in both gaseous and particulate forms based on their volatility or the prevalent ambient conditions. Low molecular weight PAHs (SPAH) like naphthalene were more volatile and existed as gas while the heavier weight PAHs (LPAH) like e.g., benzo[a]pyrene existed primarily in particulate form and these were of significant concern to human health [30]. Many PAHs and their metabolites are classified as carcinogenic, probably carcinogenic, or possibly carcinogenic to humans, as well as teratogenic and mutagenic [31],[32]. Studies have shown that ambient PAHs levels correlate with cooking events, eventually demonstrating cookstove emissions were an important source of PAHs in both indoor and outdoor air [33]. In a study by Leavey A. et al, the PAH emissions were least for solid fuels' combustion of pellets and largest for combustion of coal through all phases of cooking including, start up, steady state and extinguishing phase [33].

Decreasing the moisture content of wood induced the biomass to possess higher heating rate. This accelerated the pyrolysis and released a volatile compound rapidly through nucleation, condensation and coagulation. This study postulated that IC with increased heating rate would cause an increase in the formation of free radicals including LPAH and its health effects [33]. This brings into notice a debatable point about HAP. Dried biomass or wood used for better fuel efficiency may lead to increased release of PAH, causing detrimental health effects with concurrent decrease in emission of particulate matter and organics like Chloride, Ammonium, Nitrate and Sulfate. Further, the study found that the cumulative PAH released with the different phase of combustion times and the levels of PAH still remained high for the dried biomass [33]. Benzo(a)pyrene (B(a)P), is the most widely studied PAH and is known to be a carcinogen. Coal emissions high in (B(a)P could cause lung cancer [34]. Though ﬂuoranthene PAH in itself was not carcinogenic or harmful, it increased the carcinogenicity of (B(a)P [31].

The risk of mutagenicity related to PAHs resulted from formation of active intermediates which bound to critical positions in the DNA, RNA and proteins [31]. Even though the quantity of these intermediate PAHs was less than 10% of the parent PAH compound, these intermediates had nine times higher mutagenic potential as compared to their corresponding parent compound. Consequently, measuring these intermediate PAHs was critical in evaluating HAP related to cookstoves [35]. Most of the intermediate PAHs were associated more closely with finer particles compared to the parent PAH because of lower vaporization, diffusive ability, differences in absorption, adsorption, degradation reaction rate and gas-particle transformation rate [36]. Eventually these fine and ultrafine particles increased the exposure risk to the derivative PAHs since they penetrated deeper into lung areas and being compounds with more redox potential, elicited more inflammation and oxidative stress [37].

Long-term exposure to PAHs could cause lung cancer, bladder cancer, cataracts, liver damage, and neural tube defects [32],[34],[38]. A literature review by Shen G. concluded mutagenic emissions related to cooking are positively correlated with PM_2.5_ and PAHs emissions [39]. Another study found that installing IC reduced PAH exposure, measured by urinary hydroxylate PAH metabolites (OH-PAHs) by 19–52% [40]. Similar study evaluating the adoption of modern gasiﬁer burners found the IC to reduce personal inhalation exposure to intermediate PAHs derivatives significantly as compared to burning wood in improved brick stoves [41].

PAH is a novel biomarker for HAP. Few studies have PAH markers included in IC HAP or health related studies. The effect of IC on PAH emission is not clear with some studies showing benefit while some showing harm. PAH with its resultant mutagenic potential could be used to assess the carcinogenic effect of HAP. When measured in a study, both parent and intermediate PAH should be measured. PAH measurement may be more beneficial in studies of modifier gasifier stoves and stoves using some form of dried biomass.

In addition to the different types of emissions, there are various external factors that affect emissions related to HAP. Below we briefly describe the results of this findings.

### 3.4. Ventilation

Ventilation could be assessed by number of doors, windows, sides covered, presence or absence of roof, materials used for building and the air exchange rate in each kitchen area [42]. PM_2.5_ produced by cooking, was released partially to external environment either directly through chimney or indirectly by natural ventilation like windows and doors. For IC with a chimney, an additional direct pathway of exﬁltration for PM existed through chimney ductwork, increasing the exfiltration of PM by an average of 56%, though there was no significant difference within the newer cookstoves. Chimney exhaust losses (direct ventilation) of PM could be calculated as a difference in average indoor PM concentration measured using the IC with and without a chimney [43]. Contrarily improperly constructed chimneys restricted airflow or negatively impact the air/fuel ratio for efficient combustion causing increased PM_2.5_ concentration [42].

Improving ventilation in the rural household with additional doors and windows in kitchens would decrease concentrations of PM_2.5_ and CO in the household air [44]. Exfiltration of PM with traditional cookstoves is about 26% through natural ventilation routes like windows, doors and cracks in walls [3]. A study by Medgyesi DN et al., showed signiﬁcant regression trends of association between suppressed pulmonary function and increased HAP in the presence of inadequate ventilation demonstrating the importance of ventilation in HAP [42]. Women tend to carry their children when cooking and expose them to the acute ill effects of HAP without their knowledge. Community education about the importance of ventilation in decreasing HAP related to cookstove is an important aspect of public health to prevent health effects related to HAP [45]. In a behavioral interventional study, counselling study participants about burning wood outdoors when possible, and using two forms of ventilation during peak emission hours, observed there was a net median reduction associated with the intervention including PM_10_ reduction by 57%, CO reduction by 31% and CO concentration in the child residing in the study intervention household by 33% [46]. Similar culturally accepted interventions to improve ventilation should be advised to decrease HAP hazards in children [42]. Further, in most developing countries, the health benefits gained by improved cookstoves may be attenuated by the overall environmental pollution. Indirect risks associated with HAP exposure from traditional cooking methods extends beyond health, affecting livelihood, productivity and further exacerbating poverty [47].

Ventilation factor is seldom reported in IC studies. Though most IC studies enroll houses with similar layout for decreasing the confounding effect of ventilation. Seasonality also affects ventilation. Additionally, since there is no uniform way of measuring ventilation; some form of standardizing tool may be needed to measure ventilation for e.g ventilation score or ambient PM_2.5_ exfiltration rate to be done at regular intervals during IC studies. Thus, ventilation is a major confounder in studies related to IC and should be taken into account when planning a study on HAP related to cookstoves [48]. More objective methods to assess ventilation in research studies are needed. Further, studies are needed to assess the effect of shifting indoor air pollution to ambient air pollution. Developing estimates for the PM fraction exiting to the external environment from households in random samples during IC study period would help reduce uncertainty about the health benefits derived by IC [43].

### 3.5. Fuel type

Solid fuels included coal, charcoal, wood, straw/shrubs/grass, agricultural crop, or animal dung [49]. Insufficient fuel supply led to stove stacking in some studies. Stove stacking is wherein IC were added to the traditional stoves as a parallel cooking device when the IC malfunctioned, was unrepairable, irreplaceable and during periods of fuel shortage for IC [50]. The durability of IC was increased by promoting good IC safety profile, accessible good servicing and repairing without much specialized tools. Fuel type also affected the stove design and maintenance. Research on better fuel efficiency led to development of IC using natural draft or forced draft for cooking. Forced draft IC used processed biomass fuels in the form of pellets. Though these ICs provided superior performance, their higher initial cost and limited fuel distribution networks remained a major challenges for their widespread use [26]. On the other hand, forced draft gasifier stoves needed electric fan or some form of continuous air supply and specialized solid fuels to improve combustion and emission performance Most of these stoves did not require fuel refeeding during the cooking process which has its own associated risk. Dung cakes and agricultural residues resulted in higher emissions than the fuelwood; densified briquettes had lower emission as compared to loose fuels [26]. The amount of fuel used per weight of food cooked and the cooking time varied with type of IC, fuel used and the type of food being cooked [51]. Biogas digesters seemed a good feasible fuel option for resource limited countries since they were considered alternative sustainable energy source that help reduce HAP associated with cooking, provide fertilizer for reuse and work as a sustainable waste disposal. In general, households are perceived as units for installation, however, more research is warranted for community biogas digesters. Thus biogas could provide cleaner fuels to these communities concomitantly to curtail solid fuel usage [27],[52]. This would lead to improved health benefits to the community.

An analysis of ten low- and middle-income countries by Arku et al. found solid fuels to be the primary cooking fuel in forty-six percent of women. Further stratification showed 73% of the women were from rural areas and 23% from urban areas. Thus, solid fuels were predominantly used in rural homes. By fuel categories in this subset of charcoal and wood users, there was 0.67 mmHg and 0.51 mmHg higher systolic BP than electricity users respectively, but the difference was non-significant for systolic BP between gas and electricity users. This effect persisted with stratification by rural-urban location [2]. There is also a higher mortality noted among people using wood as compared to electricity with similar risk for ischemic heart disease related mortality. And further stratification by sex, showed a potential trend for increased risk for both all-cause and cardiovascular disease mortality in women compared with men [53]. Initial studies, including one from India emphasized earlier research for IC were prioritized towards fuel efficiency with intention to decrease fuel usage and deforestation. Consequently, the emissions levels associated with the IC were similar to traditional cookstoves [28]. Though the data showed the financial benefits of installing improved cookstoves with cost saved in decreased fuel usage, there were some shortcomings in the estimation because some households may use crop residues/agricultural waste instead of wood as fuels and would not contribute towards decreasing fuel cost [54].

**Table 1.** Biomass Cook Stoves Emissions and its Interaction with Improved Cookstove studies.

	Factors	Interaction with Improved Cookstove studies
Emission Factors	PM_2.5_	Important to monitor random outdoor PM_2.5_. Continuous monitoring of primary cook and random sampling of other members in household and PM_2.5_ in cooking area. 24-hour monitoring of primary cook has better correlation for health effects. Rural-urban stratification to confirm other sources of biomass combustions. More cumbersome and expensive.
	CO	Continuous monitoring of primary cook and random sampling of other members in household and CO in cooking area. Rural-urban stratification to confirm other sources of biomass combustions. Easier to measure, less expensive. In most studies, personal exposure corelate with kitchen exposure and hence could be monitored easily.
	PAH	Both parent and intermediate PAHs to be considered for measurement. Random pre- and post-cooking levels. Cumulative dose calculation to evaluate mutagenicity. Important in the setting of newer fuel studies (pellets/gasifier stoves/dried biomass IC) that may decrease PM_2.5_ and CO though increase PAH. More lab studies needed.
Cookstove Factors	Ventilation	Objective measure to standardize ventilation measurement across studies. Some form of random exfiltration testing. Hawthorne effect, subjects in study with knowledge of health effect more likely to change ventilation at home. Seasonality affecting ventilation.
	Fuel	Affects IC life and efficiency. Confirming fuel availability, access, acceptability and uniformity during the complete study period. Field standardization by random, regular average water boiling test time in IC households for ongoing IC efficacy.

Another important factor is the life of an IC i.e. the number of months of continuous use without appreciable loss of the original efficiency. Most traditional cookstoves are rebuilt several times. The ICs are mostly made of steel. The stove would be considered dysfunctional if significant deterioration occurs either in the structure or in thermal efficiency. Subsequently, good data is needed about the interaction of different biomass fuel with IC and their effects on the life of IC for good stove sustainability [54]. In the natural draft IC, the combustion of fuel is dependent on ambient conditions. Therefore, the thermal efficiency or the heat transfer efficiency is largely affected due to inconsistent air fuel ratio leading to low combustion temperature. Thus, the combustions process alternates between sooting (PM emission) or smoking, wherein both could have negative effect on either the IC or the household person doing the cooking [26]. HAP effect by fuels is one of the most volatile factors in IC study. Fuel supplies affect stove stacking behavior; smoking and sooting behavior of fuel affect the life of the IC which may not be same as testing in controlled settings. Water boiling test standardizes the fuel factor in laboratory-controlled studies, however few field studies could have similar standardization. Table 1 summarizes the biomass cook stove emissions and its interaction with improved cook stove studies.

## 4. Limitations

We included reviews that assessed HAP emissions related to cooking and other activities including heating. Since one of the criteria for study inclusion was health effects of HAP that was reported separately, some of the studies meeting the objective of this review may have been missed. The effect on emission within the different forms of fuels and IC was not assessed. We did not assess the quality of the articles included. We did not analyze in depth about the type of food, utensils used, geographical areas of the world and seasonality affecting HAP.

## 5. Conclusions

There is increasing use of IC to mitigate the HAP health effects. Considerable number of studies are focusing on the efficacy of IC. Because of decreased effect size of IC as health intervention, large sample size is required to assess the health outcomes of IC as intervention. Because of the significant logistic requirement, most studies are designed as pre-post intervention models as compared to randomized studies. Hence, there is a need for better comparability within the IC studies. Taking various emission and factors affecting it into account would facilitate uniformity and comparability of study results. This would further help in establishing the efficacy of IC for health mitigation and improve its uptake by public health policy. Similarly, community and health education about the health effects of HAP is important to increase IC and cleaner fuel uptake amongst communities. Assessing the community local knowledge of health- issues related to cooking and standardizing IC certification is a key factor in encouraging IC use and compliance.
